# Perspective: Young Workers at Higher Risk for Carcinogen Exposures

**DOI:** 10.3389/fpubh.2022.869232

**Published:** 2022-03-16

**Authors:** Caitlin M. Sweet, Joanne M. Telfer, Alison L. Palmer, Sajjad S. Fazel, Cheryl E. Peters

**Affiliations:** ^1^Cancer Epidemiology and Prevention Research, Cancer Care Alberta, Alberta Health Services, Calgary, AB, Canada; ^2^CAREX Canada, Faculty of Health Sciences, Simon Fraser University, Vancouver, BC, Canada; ^3^Department of Oncology, Cumming School of Medicine, University of Calgary, Calgary, AB, Canada

**Keywords:** carcinogen exposure, occupational health, youth, adolescents, vulnerable workers

## Abstract

Young workers, those under the age of 25, are considered a vulnerable working population, primarily due to their increased risk of injury. In this study we investigate if young workers may also be at an increased risk for occupational exposure to carcinogens. Using the 2006 and 2016 Canadian Census of Population and previously obtained CAREX Canada data, this study aimed to identify sectors and occupations that have high proportions of young workers and where potential exists for exposure to known and suspected carcinogens. Key groups where young workers are likely at a higher risk for occupational exposure to carcinogens were identified. Our work shows that young workers in construction, outdoor occupations, and farming are key groups that warrant further investigation. These specific groups are highlighted because of the large number of young workers employed in these sectors/situations, the high number of possible carcinogen exposures, and the potential for higher risk behavior patterns that typically occur in these types of jobs. While there is no data available to develop carcinogen exposure estimates specific to young workers, it is our perspective that young workers are likely at a higher risk for occupational exposure to carcinogens. Our findings identify opportunities to improve the occupational health and safety for this vulnerable population, particularly for young construction workers, farm workers, and outdoor workers.

## Introduction

Occupational exposure to carcinogens is an ongoing challenge in Canada and around the world. It remains extremely important to consider groups that may experience more frequent or intense exposure to carcinogens and target them for cancer prevention efforts (1). For several reasons, young workers (under the age of 25) exposed to carcinogens are at an increased risk of negative health outcomes compared to adult workers. Firstly, young workers are more biologically vulnerable since they are still undergoing physical and cognitive changes (2). This is especially true for teenage workers as they are still growing physically, breathe at a faster rate, and have higher metabolic rates relative to body size (2). Secondly, when young workers are exposed to carcinogens, they have more years in their life to develop cancers with long latency periods (2). Finally, if they stay employed in a high-risk sector long-term, they are exposed for a longer duration compared to employees who start later in life. Despite this, there is a lack of available data on whether young workers face a higher level of carcinogen exposure. To our knowledge, estimates of the number of these workers exposed to occupational carcinogens do not exist. However, several studies have investigated the increased injury rates among young workers, especially young men (3, 4). Many of the factors that contribute to increased rates of injury in this population are well-documented, and it is our perspective that these factors may also increase young worker's risk of carcinogen exposure.

The purpose of this study was to characterize the potential for carcinogen exposures among young workers in Canada. We also aimed to identify key groups of young workers for further exposure assessment investigations and prevention activities.

## Reasons for Increased Injuries

Injuries caused by acute exposure to toxic substances such as cleaning compounds, solvents, alkaline corrosives, benzene, and hydrocarbons have been investigated within the young workforce, but chronic exposure to these and other harmful substances has received little attention. Long-term exposure to such substances can cause serious chronic health effects, including skin diseases, respiratory diseases, and cancer (5, 6). A national survey from the United States found 54% of workers under age 18 had worked with one or more hazardous chemicals at their job (5). Additionally, 41% of these workers reported being exposed to fumes/thick smoke, 22% to gasoline or petroleum products, 17% to solvents or paint thinners, and 11% to pesticides (5). All of these substances may contain known or suspected carcinogens; no mention was made of the levels or duration of exposure. This is a common gap in studies investigating young worker's health and safety, which makes assessing young worker's exposures to carcinogens a difficult task.

The young worker population also has unique characteristics that make them vulnerable to workplace hazards. Young worker's limited jobsite experience and perceived inability to recognize hazards makes them particularly vulnerable. Cognitive development is not complete until the mid-twenties (7), which means that many young workers lack key decision making skills. They may not be able to assess hazards correctly or comprehend the long-term implications of disease, injury, and disability (8, 9). Furthermore, even if young workers correctly identify hazards, they are less likely voice their concerns or take action. One program targeting young workers and their parents surveyed young workers who attended their training seminars and found that nearly one third of the young workers would not refuse unsafe work, which is their right as an employee (10, 11). A Canadian study involving focus groups with 39 young workers found that the majority of young workers would not leave a job due to unsafe conditions (12). If young workers do voice their concerns, it is commonly informal, and involves speaking with other co-workers and allowing collective action to occur (12).

Young workers typically obtain entry-level jobs with minimal skill requirements. These types of jobs are more likely to involve a variety of hazards due to the use of cleaning compounds, solvents, caustics, pesticides, and other chemicals (6). These jobs are often also part-time or seasonal, which can create or contribute to a weak safety culture and decrease worker's ability to gain job-specific skills and associated safe working habits (7). Labor and advocacy groups do inform young workers and parents about safety risks involved in common jobs obtained by young workers, however this information is likely low priority in the decision making process, since the jobs young workers are qualified for are already limited (6).

Unfortunately, young workers often receive inadequate orientation and safety training. The majority of young workers do not receive proper training (13). A large Canadian study found that only 20% of young women and 23% of young men reported receiving any safety orientation within their first year of work (13). SAFE Workers of Tomorrow surveyed young workers who attended their training seminars and similarly found that 31% of the 566 young workers included in the analysis were told little to nothing about hazards in their workplace (11). This lack of safety training leads to young workers having little knowledge about hazards or strategies to mitigate risk. Young workers are more vulnerable to the effects of this lack of training compared to adult workers.

The use of personal protective equipment (PPE) among young workers is also a concern. One cross sectional study from the US investigated the PPE practices and types of PPE (i.e., burn protection, eye protection, face protection, hand protection, and noise protection) used among 866 teens (aged 14–17) employed in the retail and service sector. Among those who received PPE training (and who were in contact with hazardous chemicals), only 35% used PPE. Of those who did not receive training, 26% used PPE (5). Older, more experienced workers often believe this decision stems from young workers feeling “invincible,” or not understanding the seriousness of injuries or illnesses that could occur if proper safety precautions are not taken (14). However, many young workers state that they simply have not yet developed safe working habits, or that they did not realize the task they were performing required safety equipment (14). Furthermore, the fit and style of PPE is not always properly suited for younger workers. This has caused young workers, especially young female workers, not to use PPE as it was considered bothersome and redundant (14).

## What We Did: Data Sources

We used data from the 2006 and 2016 Canadian Censuses of Population to determine the number of young workers by sector and occupation respectively. The data were available by age strata and two-digit industry code [North American Industry Classification System (NAICS) sector], as well as two-digit occupation code [National Occupational Classification (NOC)]. The proportion of young workers in each sector and occupation was calculated by comparing the percentage of young workers in these subgroups to the overall percentage of young workers in Canada. The 2016 Canadian census and the data from CAREX Canada use the same NAICS sector codes, therefore the data is related and was used to examine potential exposures within sectors. However, the 2016 Canadian Census and the data from CAREX Canada use different NOC occupation codes and could not be directly related to one another. For this reason, the 2006 Canadian census which uses the same NOC occupation codes as CAREX Canada was used to investigate potential exposures within occupations.

The labor force data was used in conjunction with carcinogen exposure data from CAREX Canada to identify the potential for exposures in young workers. Briefly, CAREX Canada is an exposure surveillance program that has developed occupational prevalence estimates for more than 45 known or suspected carcinogens (15). These estimates were created for selected known and suspected carcinogens classified by the International Agency for Research on Cancer (IARC). CAREX Canada also created an exposures-per-worker metric for each sector and occupation as an indicator of the overall presence of occupational carcinogens and work situations where multiple exposures are expected to occur. This metric sums the number of workers exposed by sector or occupation, divided by the total number of workers in that sector or occupation (15). This pre-existing CAREX Canada data was used to examine the potential for exposure to one or more known or suspected carcinogens within sectors and occupations that employed proportionally more young workers.

The CAREX Canada exposure data and young worker data is summarized for the five sectors and occupations with the greatest proportion of young workers in Tables 1, 2, respectively. These show the percentage of young workers, the three most prevalent known or suspected carcinogen exposures based on CAREX Canada estimates, and the exposure-per-worker metric for each sector and occupation.

**Table 1:** Proportion of young workers by sector and CAREX Canada exposure data, 2016.

**Sector**	**Proportion of sector made up of young workers (%)**	**Most prevalent known or suspected carcinogen exposures**	**Exposures-per-worker metric**
Accommodation and food services	39.0%	Night shift work Polycyclic aromatic hydrocarbons (PAHs) Solar radiation	0.35
Retail trade	28.1%	Night shift work PAHs Benzene	0.29
Arts entertainment and recreation	27.9%	Solar radiation Night shift work Chloroform	0.34
Administrative and support, waste management and remediation services	14.3%	Solar radiation Night shift work Diesel engine exhaust	0.37
Agriculture, forestry, fishing and hunting	13.6%	Solar radiation Diesel engine exhaust Wood dust	1.07

**Table 2:** Proportion of young workers by occupation and CAREX Canada exposure data, 2006.

**Occupation**	**Proportion of occupation made up of young workers (%)**	**Top exposures**	**Exposures-per-worker metric**
Retail salespersons, sales clerks, cashiers	42.1%	Night shift work Polycyclic aromatic hydrocarbons (PAHs) Solar radiation	0.24
Chefs, cooks, and servers	41.1%	Night shift work PAHs Formaldehyde	0.52
Sales and service occupations	32.2%	Night shift work PAHs Solar radiation	0.21
Trades helpers, construction and transportation laborers	27.6%	Solar radiation Silica Night shift work	1.20
Occupations unique to primary sector	20.8%	Solar radiation Diesel engine exhaust Night shift work	1.22

In 2016, 13.5% of Canada's workforce was made up of young workers (16). Sectors with a higher proportion of young workers include accommodation and food services (39.6%), retail trade (28.1%), arts entertainment and recreation (27.9%), administrative and support, waste management and remediation services (14.3%), and agriculture, forestry, fishing and hunting (13.6%). Among the sectors with the highest proportion of young workers, the agriculture, forestry, fishing and hunting sector has the largest exposures-per-worker metric (1.07).

In 2006, young workers represented 15.8% of the Canadian workforce and more than 40% of workers in some occupations (16). Occupations with a higher proportion of young workers include retail salespersons, sales clerks and cashiers (42.1%), chefs, cooks and servers (41.1%), sales and service occupations (32.2%), trades helpers, construction and transportation laborers (27.6%), and occupations unique to primary sector (20.8%). Trades helpers, construction and transportation laborers, as well as the occupations unique to primary sector both have exposures-to-worker metrics greater than one (Table 2).

Supplementary Tables A.1, A.2 online show the proportion of young workers and exposures-per-worker metrics for all two-digit sectors and occupations, respectively.

## Discussion

Young workers comprise a significant proportion of Canada's workforce, with more than 30% of the workforce in some sectors and more than 40% in some occupations (16). These workers are known to have an increased risk of injury while on the job (17). Some of the risk behavior patterns leading to increased injury rates are reasonably likely to lead to increased carcinogen exposure. For example, young worker's lower ability to assess hazards correctly and understand the long term implications of disease, injury, and disability compared to matured adults is a factor that causes higher injury rates (8, 9). These characteristics could also make young workers less able to assess the seriousness or consequences of carcinogen exposures. In turn, even if they are assessed correctly, young workers are less likely to turn down unsafe tasks (i.e., working with carcinogens) or leave an unsafe job (10–12). Additionally, the majority of young workers receive inadequate safety training or no safety training at all (11, 13). This leads young workers to know little about the hazards of their workplace and a lack of understanding of the appropriate ways to reduce hazardous exposures. Finally, PPE is a major concern when investigating the possible correlation between increased injuries and carcinogen exposures. Since studies have found that young workers are less likely to know if a task requires PPE, less likely to remember to wear PPE and less likely to use PPE because it does not fit them properly (14), they are less likely to be protected against hazardous exposures.

While there is no available data to develop carcinogen exposure estimates specific to young workers, we have identified groups of young workers that are likely at a higher risk for occupational exposure to carcinogens. This includes young construction workers, farm workers, and outdoor workers. These groups were determined to be high priority by considering: (a) the risk behavior patterns of young workers; (b) the sectors where a large proportion of young workers are employed; and (c) the sectors with a higher number of potential occupational exposures.

### Young Construction Workers

Although a modest proportion of the construction sector is made up of young workers (11.4%), it is still a common sector for this population, with well over 150,000 young Canadians employed in both 2006 and 2016 (16). This is especially true for young male workers; in 2016, 11% of all young Canadian male workers were employed in the construction sector.

The construction sector has long been known as a hazardous sector for young workers (18) and has a large number of associated carcinogenic exposures. In fact, CAREX Canada reports that this sector has the highest exposures-per-worker value (at 1.11). WorkSafeBC identified construction as a common sector where young workers are employed that has the potential for exposure to asbestos, silica dust, lead, and other chemicals (19). Both silica and asbestos are classified by IARC as Group 1 (*carcinogenic to humans)* and inorganic lead is classified as Group 2A (*probably carcinogenic to humans*) (20). Asbestos is a particularly concerning exposure as there is no “safe” level of exposure, and the cancer risk increases as duration and level of exposure increases (2). Other common carcinogens in the construction sector include solar radiation, wood dust, and diesel engine exhaust (21). Considering the higher number of potential carcinogen exposures in construction, a lack of knowledge about hazards and insufficient PPE would be of particular concern in this sector.

### Young Outdoor Workers

Many jobs that require working for extended periods outdoors are often filled by young workers. Examples of outdoor jobs typically held by young workers include ground maintenance laborers, painters, tree planters, lifeguards, construction workers, and general farm workers (16). Such jobs expose workers to solar radiation and additional hazards depending on the job type. Solar radiation has been classified by IARC as Group 1 (*carcinogenic to humans*) with an established link to skin cancer and possible links to melanoma of the eye and non-Hodgkin lymphoma (22). A 2006 study investigated the work-time sun behaviors of 1,330 Canadian outdoor workers (254 were young workers). It found that young workers were significantly more likely to spend four or more hours in the sun compared to older outdoor workers and less likely to use sun protection, such as protective clothing, sunglasses, or sunscreen (23). This is concerning as getting sunburns at a young age is more strongly correlated to melanoma compared to getting sunburns at an older age (2). Outdoor workers are also at risk of a variety of other exposures, including extreme heat or cold, noise, biological hazards such as insects, and vector-borne diseases such as Lyme disease (24, 25).

### Young Farm Workers

According to the 2016 census, 81% of young workers in primary industry work in farming (16). This may be an underestimation, because the census only includes workers over the age of 15, so family members or others under this age restriction doing farm work would not be included. The most prevalent occupational carcinogen exposures in the farming sector are solar radiation, diesel engine exhaust, and glyphosate (26). The CAREX Canada exposures-per-worker metric of 1.07 for the agriculture, forestry, fishing, and hunting sector is an underestimate, as it only includes four pesticides that had available data (2,4-D, glyphosate, chlorothalonil, and pentachlorophenol), and farm workers can potentially be exposed to a wider range of pesticides. Pesticide exposure at a young age may increase the risk of childhood leukemia and can cause neurological and behavioral development issues (2, 27, 28). A community-based survey found adolescents had significantly lower pesticide knowledge scores compared to adult agriculture workers. The same survey found that only 14% of the teens reported receiving any pesticide safety training, despite the fact that 86% of them reported being exposed to pesticides (9).

## Limitations

There are a few limitations to our approach that should be considered. While using the Canadian census is the best available approach to capturing the population of young workers, it has limitations. Young workers often hold temporary or seasonal jobs and some of these jobs may not be accounted for using census data. The census asks about an individual's job during a certain week of the year, and if the individual did not work that week they are to answer questions based on their job of longest duration in the past year (29, 30). Furthermore, the census only asks about workers over the age of 15, meaning any employee under this age restriction would not be included in the dataset. There are few provinces that allow employment of children under the age of 14, but certain provinces have exceptions for select sectors (31). Some provinces exempt or alter child labor laws for children working on family farms, meaning the actual number of young workers within the agriculture sector may be higher than the values reported. This is concerning as agriculture is a sector with potential exposure to several known carcinogens, such as solar radiation, diesel engine exhaust, pentachlorophenol, and other pesticides (26).

Additionally, it is important to re-iterate that CAREX Canada data available for this analysis uses the same occupation (NOC-S) and sector (NAICS) classification systems as the 2006 census. Since the 2016 census used a different occupation classification system than in 2006, CAREX Canada occupation prevalence estimates and occupation exposure per-worker-metric could not be directly compared to the 2016 young worker population. For this reason, Table 2 (occupation-based) uses 2006 young worker census data. The 2016 census used a very similar NAICS system as the 2006 census, therefore Table 1 (sector-based) presents CAREX Canada data in relation to the 2016 young worker data. Finally, we only reported on young worker's exposure to carcinogens that exist in the CAREX Canada database. Young workers could be exposed to a longer list of carcinogens that have not yet been assessed by CAREX Canada.

In conclusion, although there is a lack of available data to develop carcinogen exposure estimates for young workers, we have demonstrated the unique characteristics that make this population vulnerable may also lead to increased risk of exposure to carcinogens. This is especially true for young construction, outdoor and farm workers. However, it is important for future studies to develop accurate occupational carcinogen estimates for young workers, as they can inform relevant policies and programs to better protect this population and reduce their risk of cancer.

## Data Availability Statement

The data analyzed in this study is subject to the following licenses/restrictions: The census datasets analyzed for this study can be accessed by contacting the authors, and CAREX data can be found in CAREX Canada: An enhanced model for assessing occupational carcinogen exposure (https://oem.bmj.com/content/oemed/72/1/64.full.pdf). Requests to access these datasets should be directed to cheryl.peters@ucalgary.ca.

## Author Contributions

CP, JT, SF, and AP contributed to conception of the manuscript. CS collated the data and wrote the first draft of the manuscript. All authors contributed to various sections of the manuscript, revisions, read, and approved the submitted version.

## Funding

CAREX Canada was funded by the Canadian Partnership Against Cancer.

## Conflict of Interest

The authors declare that the research was conducted in the absence of any commercial or financial relationships that could be construed as a potential conflict of interest.

## Publisher's Note

All claims expressed in this article are solely those of the authors and do not necessarily represent those of their affiliated organizations, or those of the publisher, the editors and the reviewers. Any product that may be evaluated in this article, or claim that may be made by its manufacturer, is not guaranteed or endorsed by the publisher.

## Abbreviations

PPE: Personal protective equipment
NAICS: North American Industry Classification System
NOC: National Occupational Classification
IARC: International Agency for Research on Cancer.
