# Supplementary material for: Perspective: Young Workers at Higher Risk for Carcinogen Exposures
Source: Front Public Health. 2022 Mar 16;10:869232. doi: 10.3389/fpubh.2022.869232 (PMC8966076; doi:10.3389/fpubh.2022.869232)
Supplement: Supplementary file 1 [file Data_Sheet_1.docx]

**Supplemental Tables:**

## Table A.1: Young worker population by sector, 2016

| **Industry (NAICS 2012)** | **Workers of all ages population** | **Young worker population** | **Percentage of industry made up of young workers (%)** | **Exposures per-worker metric** |
| --- | --- | --- | --- | --- |
| *Total* | *18,268,100* | *2,472,500* | ***13.5%*** |  |
| Accommodation and food services (72) | 1,283,700 | 507,800 | **39.6%** | 0.35 |
| Retail trade (44-45) | 2,110,200 | 592,300 | **28.1%** | 0.29 |
| Arts, entertainment and recreation (71) | 379,700 | 105,800 | **27.9%** | 0.34 |
| Administrative and support, waste management and remediation services (56) | 802,400 | 114,500 | **14.3%** | 0.37 |
| Agriculture, forestry, fishing and hunting (11) | 444,700 | 60,400 | **13.6%** | 1.07 |
| Other services (except public administration) (81) | 814,700 | 95,000 | **11.7%** | 0.49 |
| Construction (23) | 1,365,000 | 155,300 | **11.4%** | 1.11 |
| Information and cultural industries (51) | 420,400 | 47,500 | **11.3%** | 0.18 |
| Manufacturing (31-33) | 1,596,600 | 137,100 | **8.6%** | 0.74 |
| Educational services (61) | 1,346,600 | 110,200 | **8.2%** | 0.06 |
| Wholesale trade (41) | 665,700 | 52,000 | **7.8%** | 0.24 |
| Public administration (91) | 1,128,900 | 86,700 | **7.7%** | 0.41 |
| Health care and social assistance (62) | 2,138,000 | 157,100 | **7.3%** | 0.27 |
| Real estate and rental and leasing (53) | 333,300 | 24,000 | **7.2%** | 0.09 |
| Professional, scientific and technical services (54) | 1,335,100 | 92,600 | **6.9%** | 0.08 |
| Mining, quarrying, and oil and gas extraction (21) | 271,300 | 18,200 | **6.7%** | 1.09 |
| Finance and insurance (52) | 790,600 | 51,700 | **6.5%** | 0.05 |
| Transportation and warehousing (48-49) | 876,100 | 55,200 | **6.3%** | 0.93 |
| Utilities (22) | 136,300 | 7,800 | **5.8%** | 0.41 |
| Management of companies and enterprises (55) | 28,800 | 1,500 | **5.1%** | 0.19 |

**Table A.2: Young worker population by occupation, 2006**

| **Occupational group (NOC-S 2006)** | **Workers of all ages population** | **Young worker population** | **Percentage of occupation made up of young workers(%)** | **Exposures per-worker metric** |
| --- | --- | --- | --- | --- |
| *Total* | *16,861,200* | *2,662,500* | ***15.8%*** |  |
| Retail salespersons, sales clerks, cashiers (G011, G211 - G311) | 1,123,500 | 472,600 | **42.1%** | 0.24 |
| Chefs and cooks, and servers (G012, G411-G513) | 550,500 | 226,100 | **41.1%** | 0.52 |
| Sales and service occupations (G013 - G016, G711 - G732, G911 - G983) | 1,473,700 | 474,900 | **32.2%** | 0.21 |
| Trades helpers, construction and transportation labourers (H811 - H832) | 402,100 | 110,900 | **27.6%** | 1.2 |
| Occupations unique to primary industry (I011 - I216) | 648,300 | 134,800 | **20.8%** | 1.09 |
| Occupations in art, culture, recreation, sport (F011 - F154) | 502,200 | 99,900 | **19.9%** | 0.14 |
| Labourers in processing, manufacturing, and utilities (J311 - J319) | 266,600 | 52,300 | **19.6%** | 0.79 |
| Clerical occupations, including supervisors (B411 - B576) | 1,733,100 | 276,200 | **15.9%** | 0.1 |
| Construction trades (H111 - H145) | 436,600 | 67,300 | **15.4%** | 1.34 |
| Childcare and home support workers (G811 - G814) | 259,700 | 36,100 | **13.9%** | 0.06 |
| Occupations in protective services (G611 - G631) | 277,200 | 36,900 | **13.3%** | 1.1 |
| Other trades occupations (H211 - H535) | 908,700 | 114,000 | **12.5%** | 1.24 |
| Technical, assisting, and related occupations in health (D211 - D313) | 478,500 | 55,400 | **11.6%** | 0.4 |
| Machine operators and assemblers in manufacturing, including supervisors (J011 - J228) | 726,100 | 79,900 | **11.0%** | 0.94 |
| Occupations in social science, government service, religion (E011 - E039, E211 - E217) | 746,300 | 69,800 | **9.4%** | 0.09 |
| Natural and applied sciences and related occupations (C011 - C183) | 1,108,100 | 95,300 | **8.6%** | 0.17 |
| Transport and equipment operators (H611 - H737) | 677,700 | 47,200 | **7.0%** | 1.74 |
| Wholesale, technical, insurance, real estate sales specialists, and retail, wholesale and grain buyers (G111 - G134) | 353,100 | 23,100 | **6.5%** | 0.02 |
| Financial, Secretarial, Admin occupations (B111 - B318) | 864,700 | 53,400 | **6.2%** | 0.02 |
| Teachers and professors (E111 - E133) | 668,000 | 37,900 | **5.7%** | 0.05 |
| Contractors and supervisors in trades and transportation (H011 - H022) | 125,200 | 5,300 | **4.2%** | 0.66 |
| Other management occupations (A111 - A392) | 1,413,000 | 59,500 | **4.2%** | 0.05 |
| Professional occupations in business/finance (B011 - B022) | 427,600 | 16,000 | **3.7%** | 0.01 |
| Professional occupations in health, nurse supervisors, nurses (D011 - D112) | 471,900 | 15,400 | **3.3%** | 0.37 |
| Senior management Occupations (A011 - A016) | 218,600 | 2,500 | **1.1%** | 0 |
